# Supplementary material for: Dual hydrogen production from electrocatalytic water reduction coupled with formaldehyde oxidation via a copper-silver electrocatalyst
Source: Nat Commun. 2023 Jan 31;14:525. doi: 10.1038/s41467-023-36142-7 (PMC9889775; doi:10.1038/s41467-023-36142-7)
Supplement: Supplementary file 1 — Supplementary Information [file 41467_2023_36142_MOESM1_ESM.docx]

Supplementary Information

**Dual Hydrogen Production from** **Electrocatalytic Water Reduction Coupled with Formaldehyde Oxidation via a Copper-Silver Electrocatalyst**

Guodong Li,^1^ Guanqun Han,^1^ Lu Wang,^2^ Xiaoyu Cui,^1^ Nicole K. Moehring,^3,4,5^ Piran R. Kidambi,^3,4,5^ De-en Jiang,^2,3^* and Yujie Sun^1^*

1. Department of Chemistry, University of Cincinnati, Cincinnati, Ohio 45221, USA
2. Department of Chemistry, University of California Riverside, Riverside, California 92521, USA
3. Department of Chemical and Biomolecular Engineering, Vanderbilt University, Nashville, Tennessee 37212, USA
4. Interdisciplinary Graduate Program in Materials Science, Vanderbilt University, Nashville, Tennessee 37235, USA
5. Vanderbilt Institute of Nanoscale Science and Engineering, Nashville, Tennessee 37212, USA

*E-mail: yujie.sun@uc.edu; de-en.jiang@vanderbilt.edu

**Inventory of Supplementary Information**

Supplementary Figure 1-4 and 20 are related to the Fig. 2.

Supplementary Figure 5-6 are related to the Fig. 3.

Supplementary Figure 7-15 and Supplementary Table 1-2 are related to Fig. 3 and Fig. 4.

Supplementary Figure 16 is related to Fig. 5.

Supplementary Figure 17-19 and Supplementary Table 3 are related to Fig. 6.


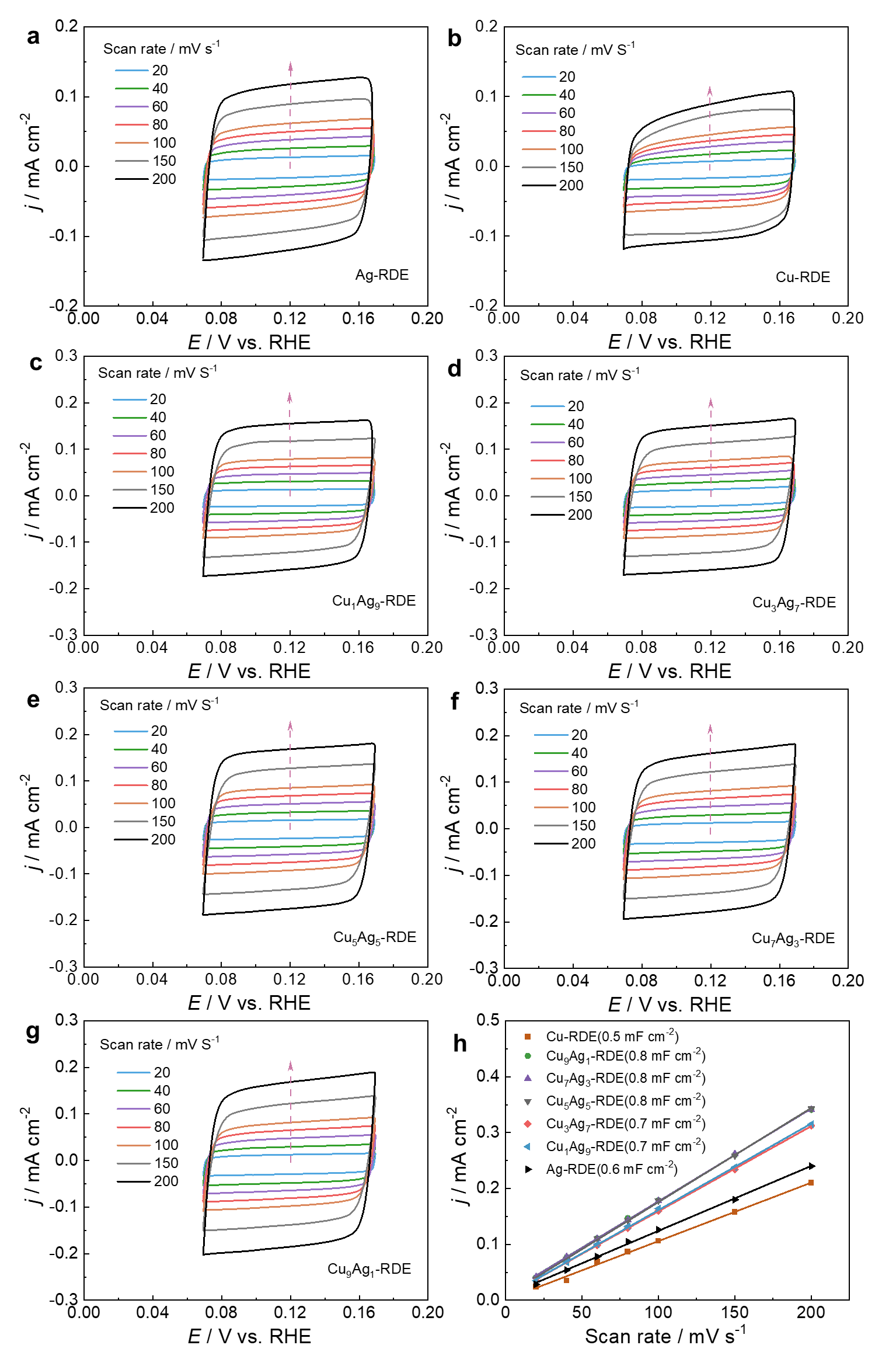


**Supplementary Fig. 1** **a**-**g** CV curves for the Cu/RDE, Ag/RDE, and Cu_x_Ag_10-x_/RDE electrodes were collected in a non-Faradaic region with various scan rates ranging from 20 to 200 mV s-^1^ at potentials between 0.07 V and 0.17 V vs RHE in 1.0 M KOH under Ar. (**h**). Electrochemical double-layer capacitances (Cdl) values of Cu/RDE, Ag/RDE, and Cu_x_Ag_10-x_/RDE electrodes based on CV curves in (**a**-**g**).


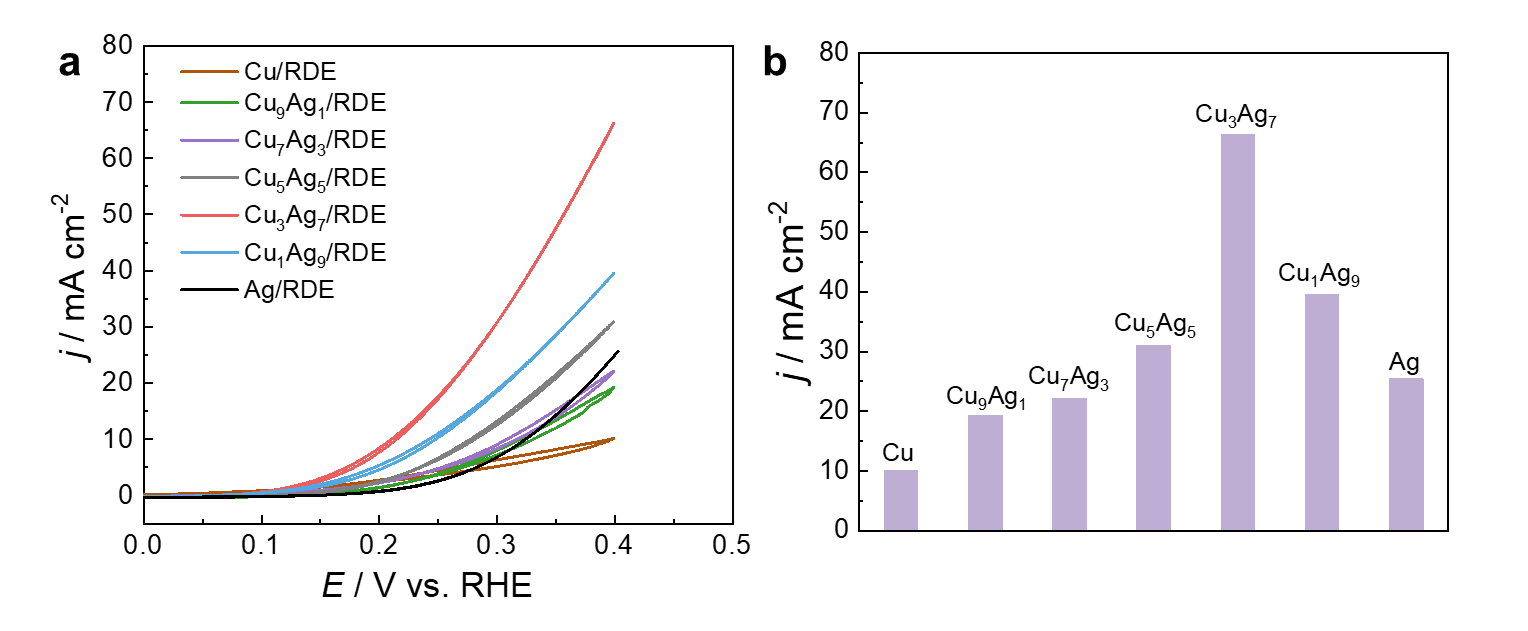


**Supplementary Fig. 2 a** CV curves of Cu/RDE, Ag/RDE, and Cu_x_Ag_10-x_/RDE electrodes in 1.0 M KOH in the presence of 0.6 M HCHO collected at 1500 rpm and 10 mV/s under Ar. **b** Current densities of Cu/RDE, Ag/RDE, and Cu_x_Ag_10-x_/RDE electrodes in 1.0 M KOH with 0.6 M HCHO at 0.4 V_RHE_ collected at 1500 rpm and 10 mV/s under Ar.


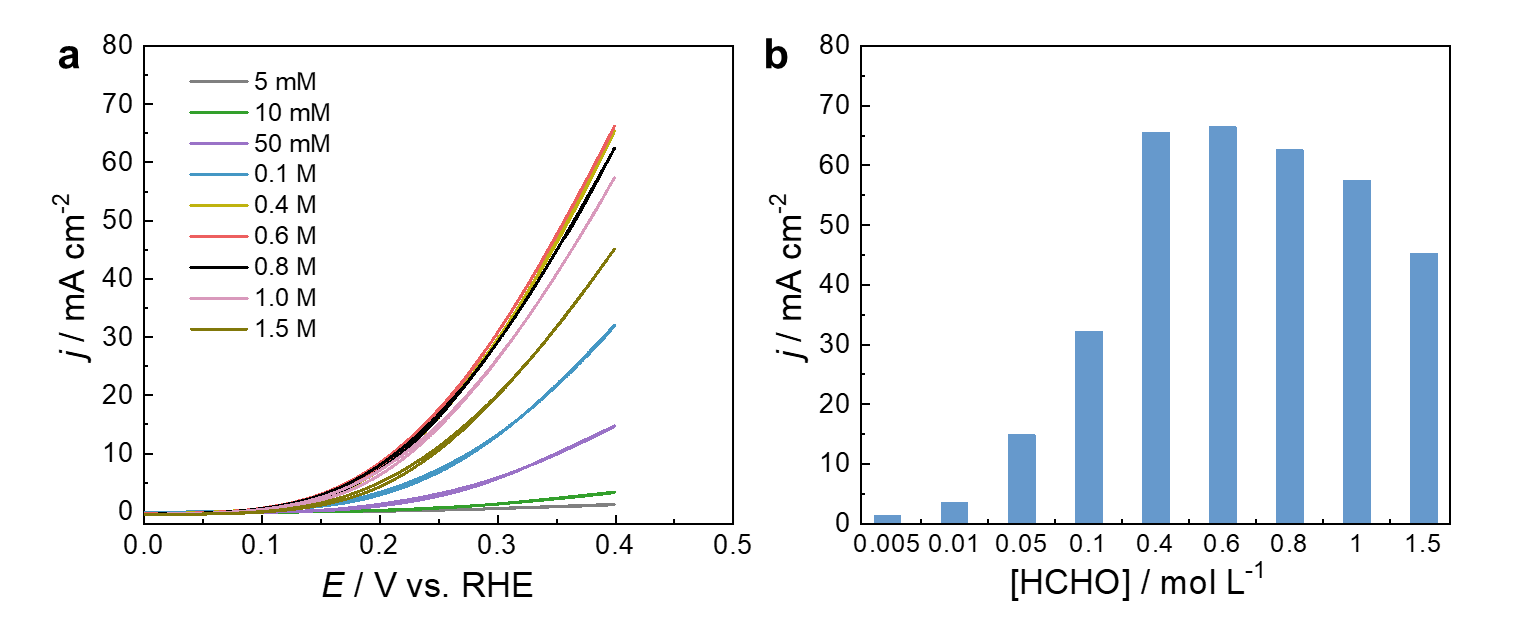


**Supplementary Fig. 3 a** CV curves of Cu_3_Ag_7_/RDE in 1.0 M KOH with different concentrations of HCHO collected at 1500 rpm and 10 mV/s under Ar. **b** The current density values of Cu_3_Ag_7_/RDE in 1 M KOH with different concentrations of HCHO at 0.4 V_RHE_ collected at 1500 rpm and 10 mV/s under Ar.


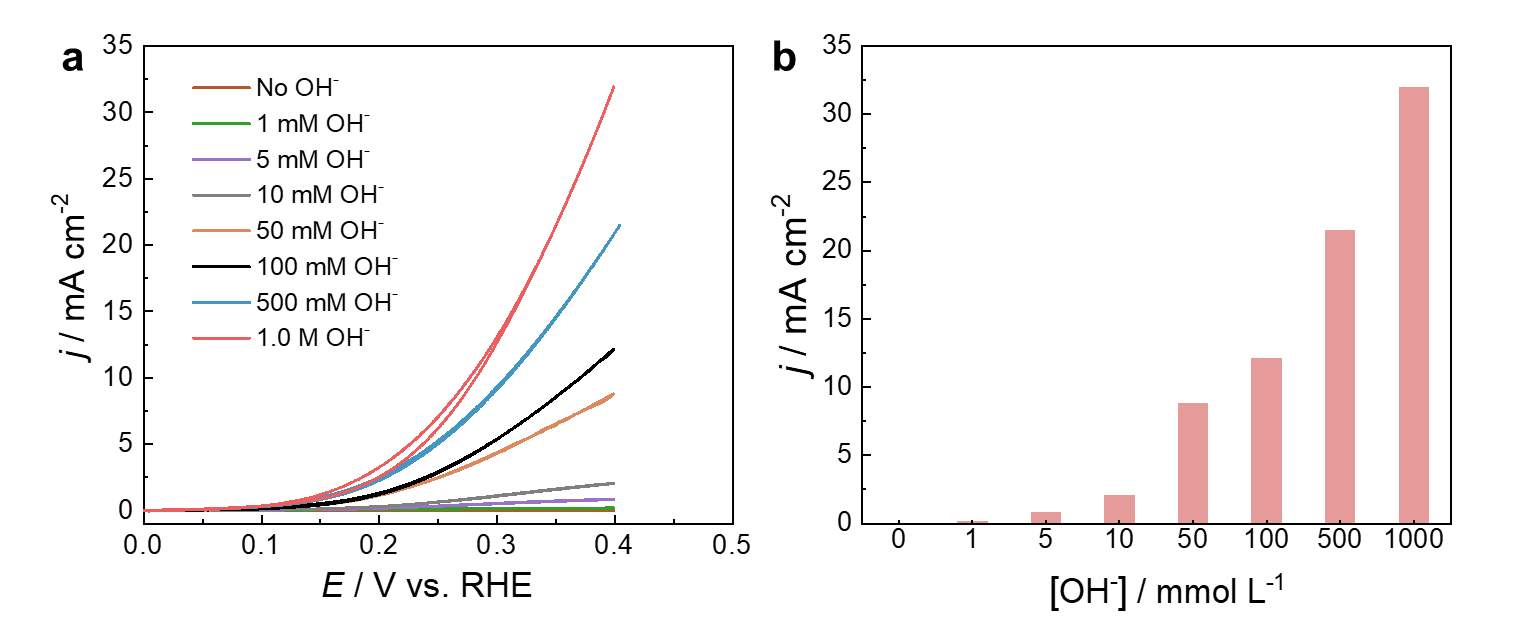


**Supplementary Fig. 4 a** CV curves of Cu_3_Ag_7_/RDE in 1.0 M anion concentration anolyte containing different OH^-^ concentrations in the presence of 0.6 M HCHO collected at 1500 rpm and 10 mV/s under Ar. **b** The current density values of Cu_3_Ag_7_/RDE in 0.6 M HCHO with different concentrations of OH^-^ at 0.4 V_RHE_ collected at 1500 rpm and 10 mV/s under Ar.


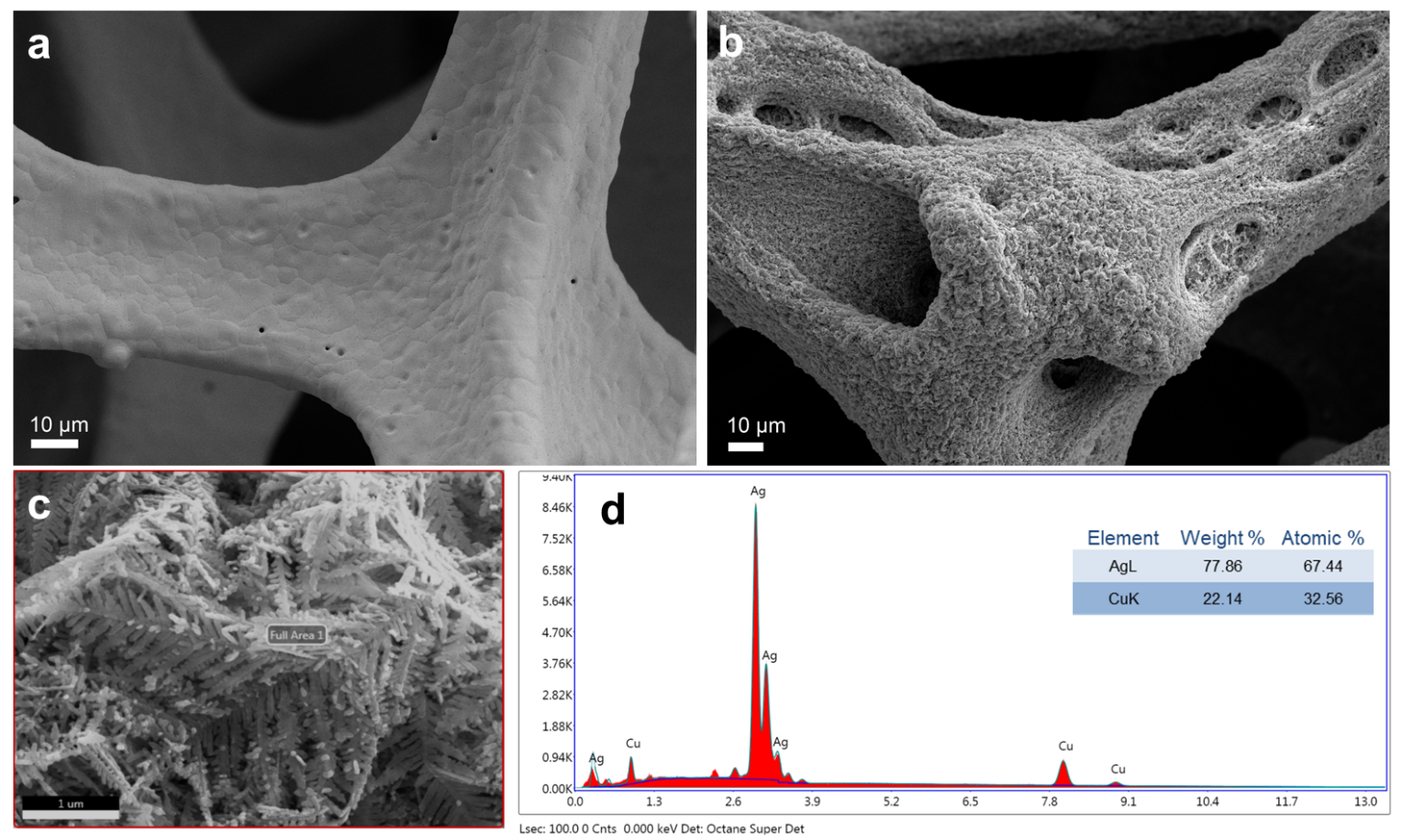


**Supplementary Fig. 5 a** The scanning electron microscopy (SEM) image of a pristine copper foam. **b** SEM images of Cu_3_Ag_7_/CF; **c-d** Energy-dispersive X-ray spectroscopic (EDX) analysis of Cu_3_Ag_7_/CF.


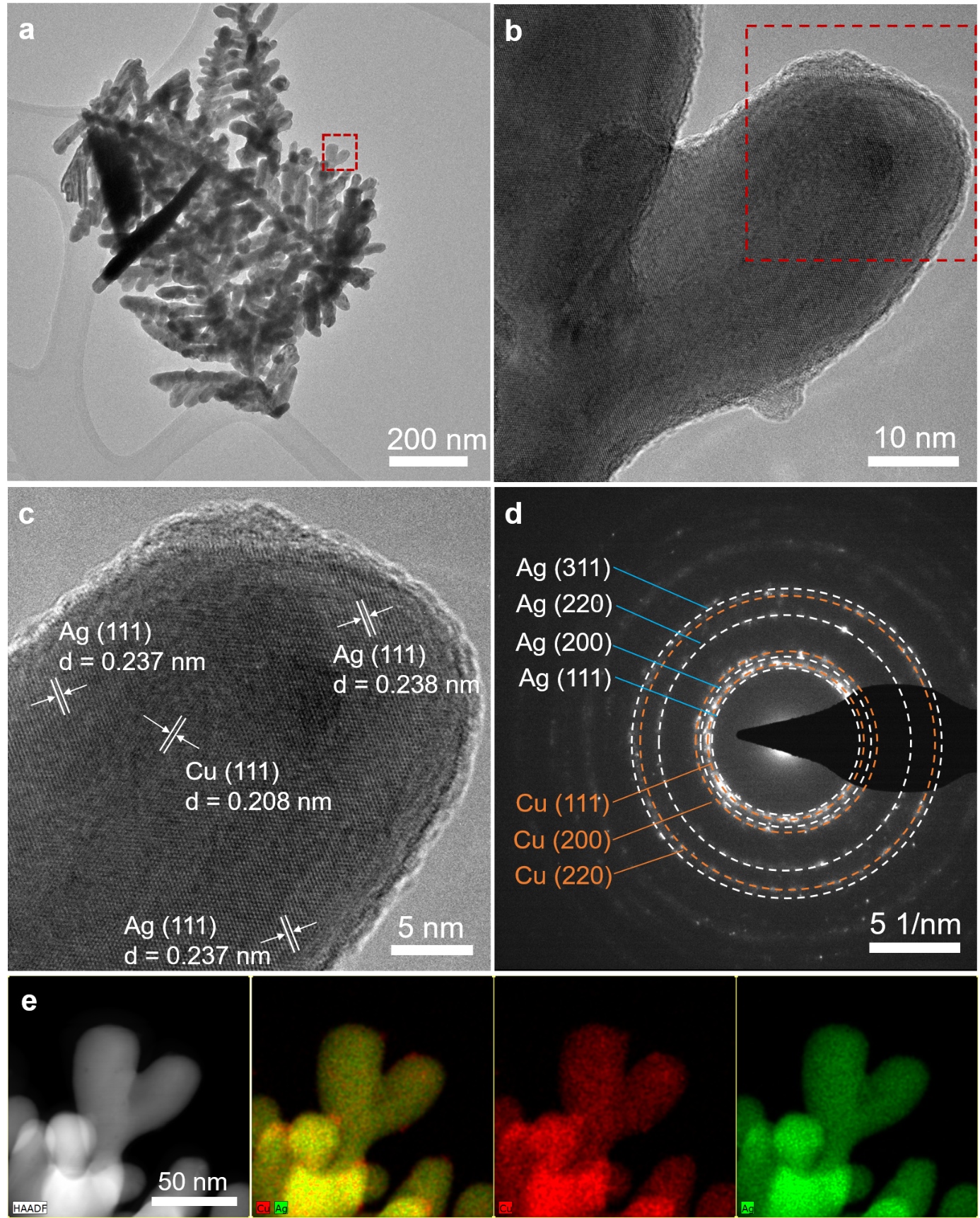


**Supplementary Fig. 6** **a** TEM image of Cu_3_Ag_7_/CF dendritic structure. **b-c** HR-TEM images of Cu_3_Ag_7_/CF. **d** SAED of Cu_3_Ag_7_/CF. **e** HAADF image and EDX mapping of Cu_3_Ag_7_/CF.


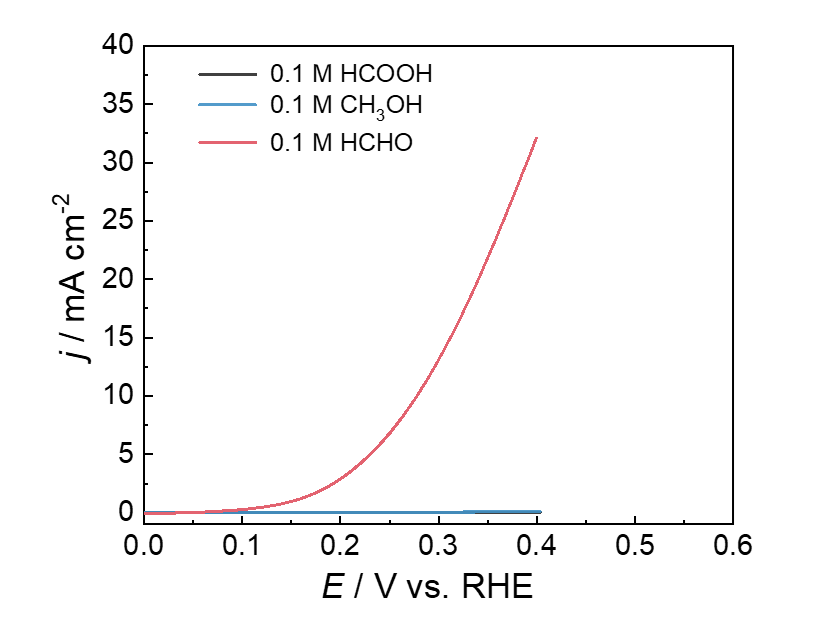


**Supplementary Fig. 7**. LSV curves of Cu_3_Ag_7_/RDE in 1.0 M KOH upon the addition of 0.1 M HCOOH (black), 0.1 M CH_3_OH (blue), and 0.1 M HCHO (red) collected at 1500 rpm and 10 mV/s under Ar.


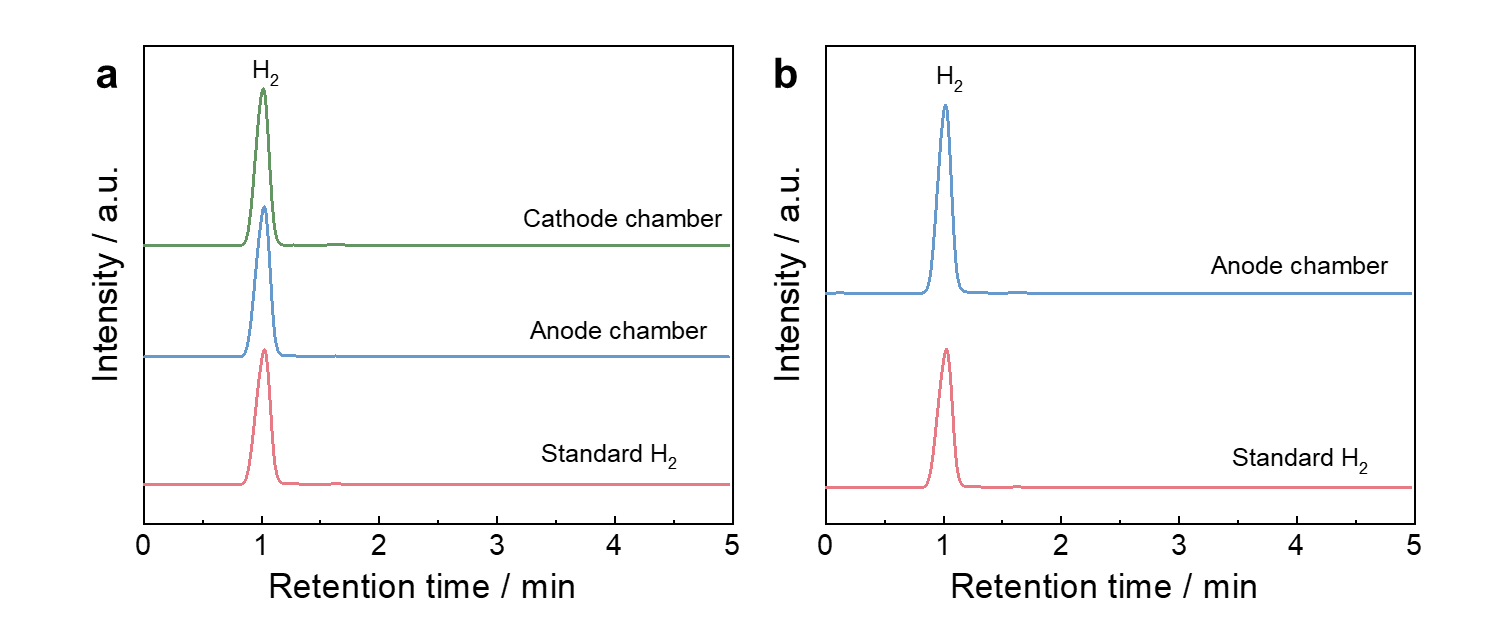


**Supplementary Fig. 8** **a** The gas chromatography of gas products collected from each cathode and anode chamber in a two-electrode electrolyzer consisting of Cu_3_Ag_7_/CF anode and Ni_3_N/Ni/NF cathode at a cell voltage of 0.6 V, in which 1.0 M KOH was used as the catholyte and 1.0 M KOH with 0.6 M HCHO as the anolyte. **b** The gas chromatography of gas products collected from the anode chamber in a two-compartment electrochemical cell using Cu_3_Ag_7_/CF as the working electrode at 0.4 V_RHE_, in which 1.0 M KOH was used as the catholyte and 1.0 M KOH with 10.0 g/L paraformaldehyde as the anolyte.

**
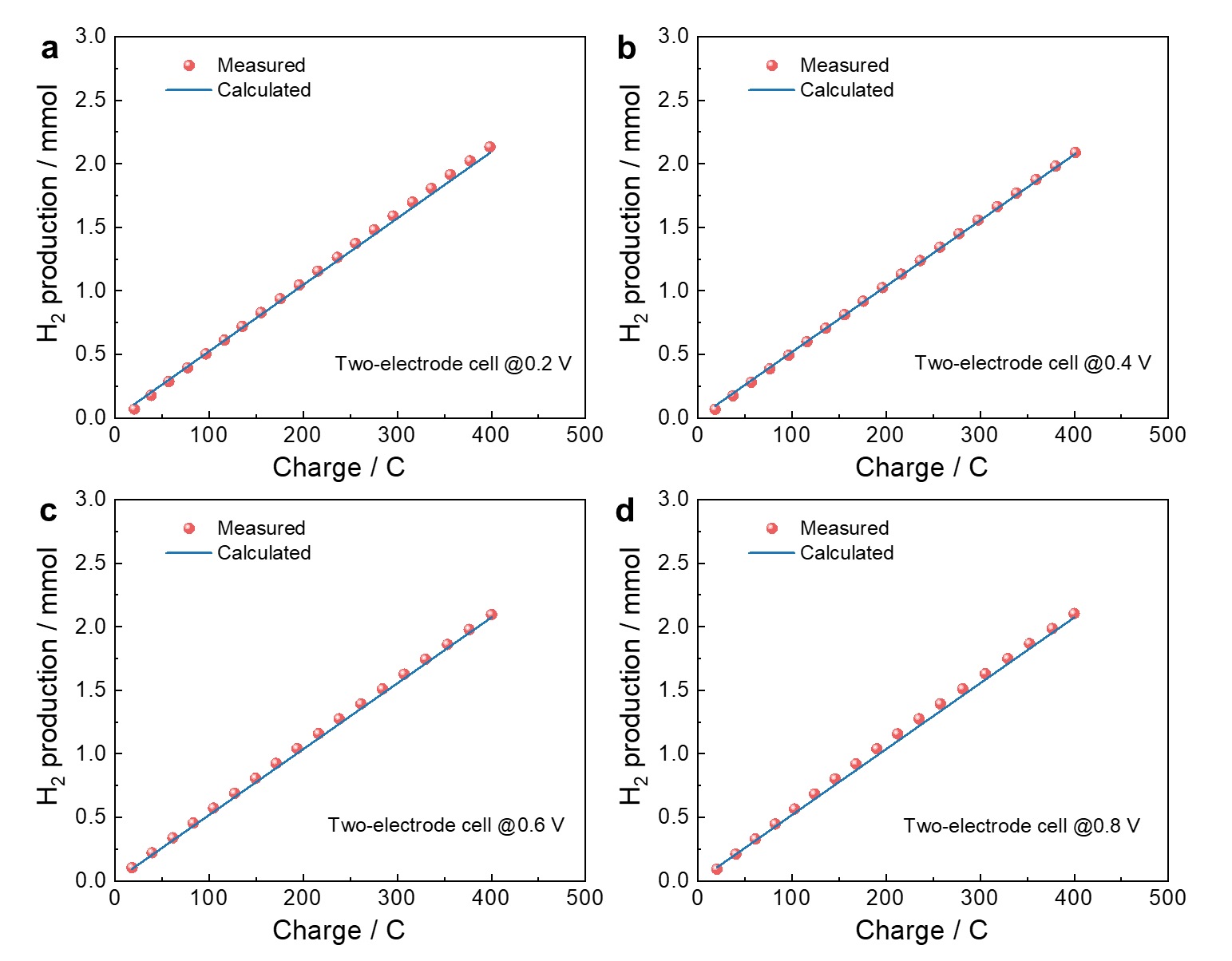
**

**Supplementary Fig. 9** **a-d** Comparison of the experimentally measured H_2_ amounts with the theoretical H_2_ amounts along the passed charge for the anode chambers during each electrolysis with different voltage inputs (0.2 – 0.8 V) in a two-electrode electrolyzer consisting of Cu_3_Ag_7_/CF anode and Ni_3_N/Ni/NF cathode, in which 1.0 M KOH was used as the catholyte and 1.0 M KOH with 0.6 M HCHO as the anolyte.

**
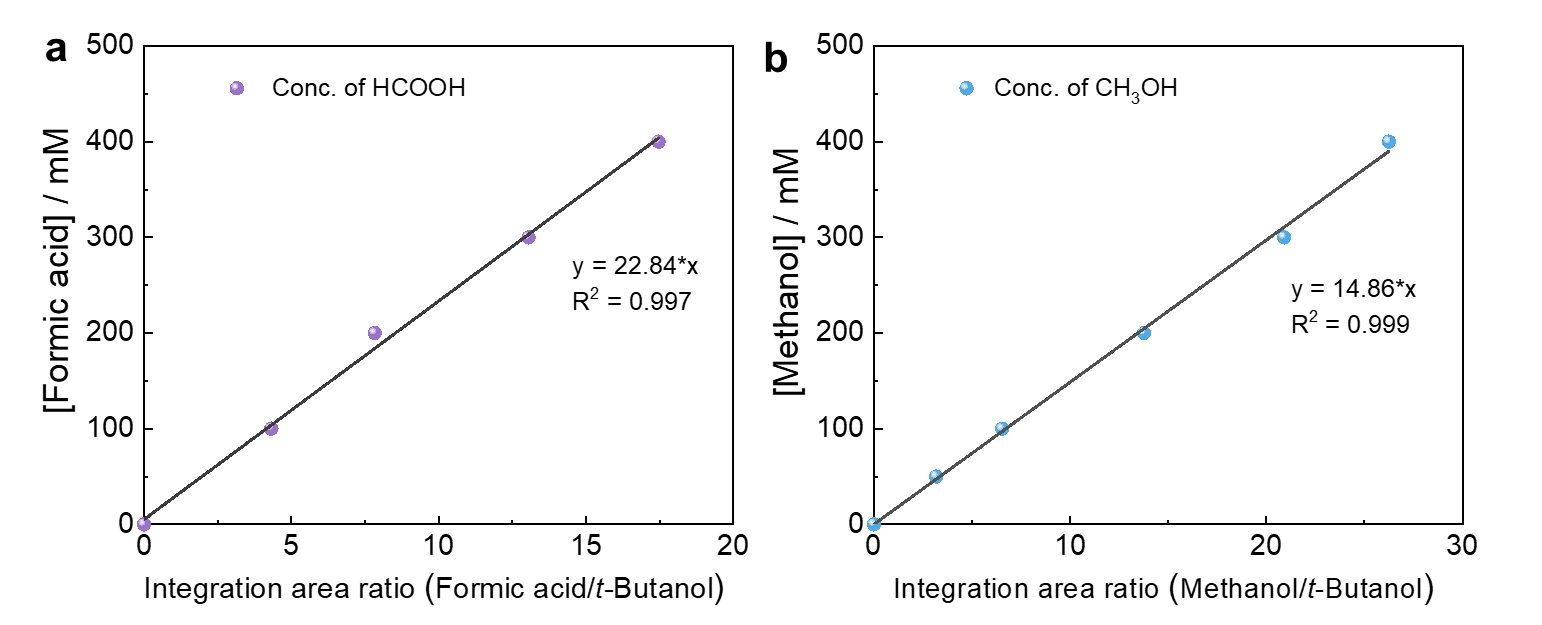
**

**Supplementary Fig. 10** Calibration curves for the ^1^H NMR quantification of (**a**) formic acid and (**b**) methanol using *t*-butanol as an internal standard.


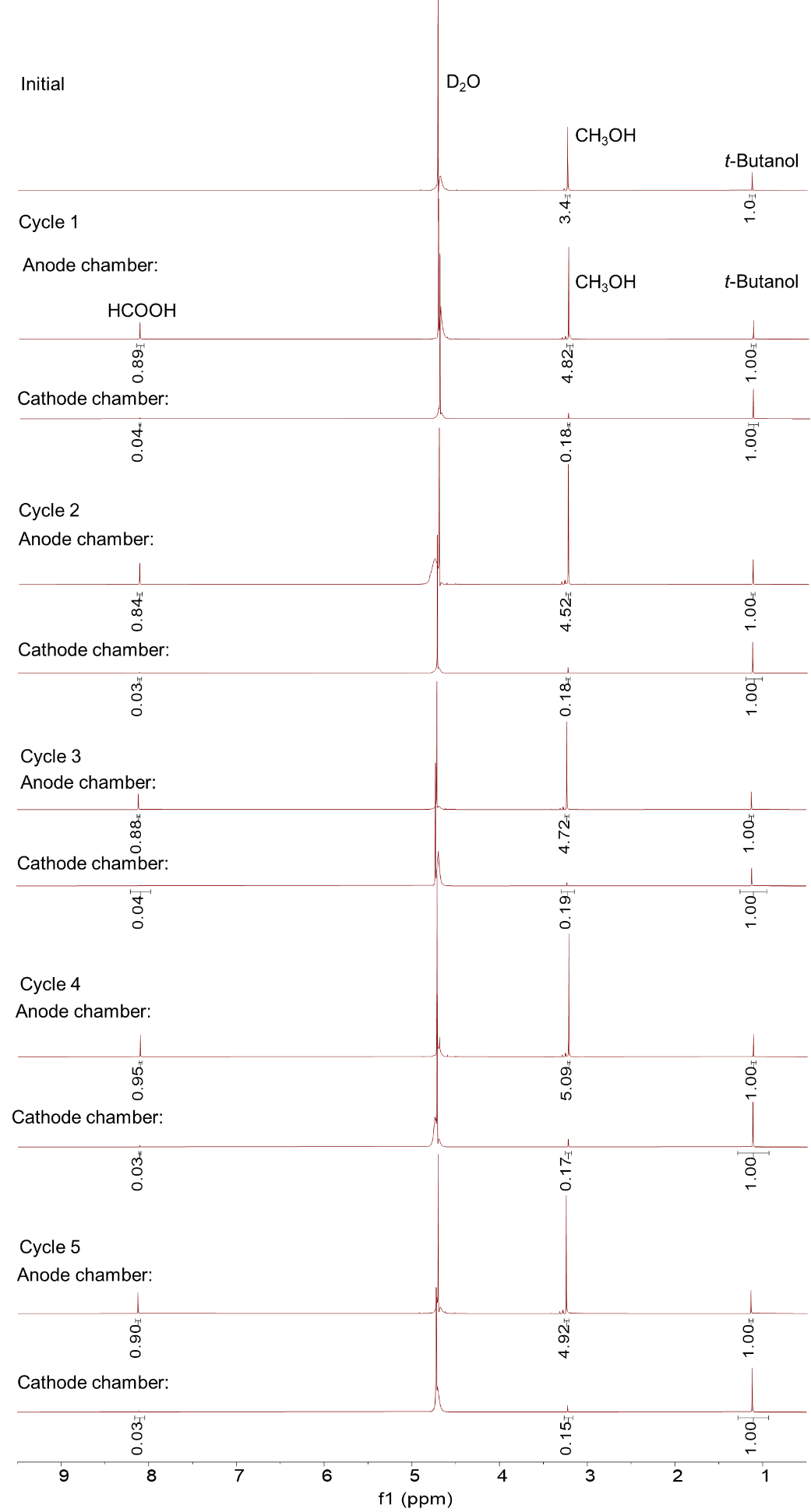


**Supplementary Fig. 11** The ^1^H NMR (D_2_O, 400 MHz) of organic products in the liquid phase of the anode and cathode chamber at five consecutive 1 h controlled-current electrolysis (150 mA) in a two-electrode electrolyzer using the Cu_3_Ag_7_/CF and Ni_3_N/Ni/NF couple but fresh electrolyte for each cycle. The anolyte was 1.0 M KOH with 0.6 M HCHO and the catholyte is 1.0 M KOH.


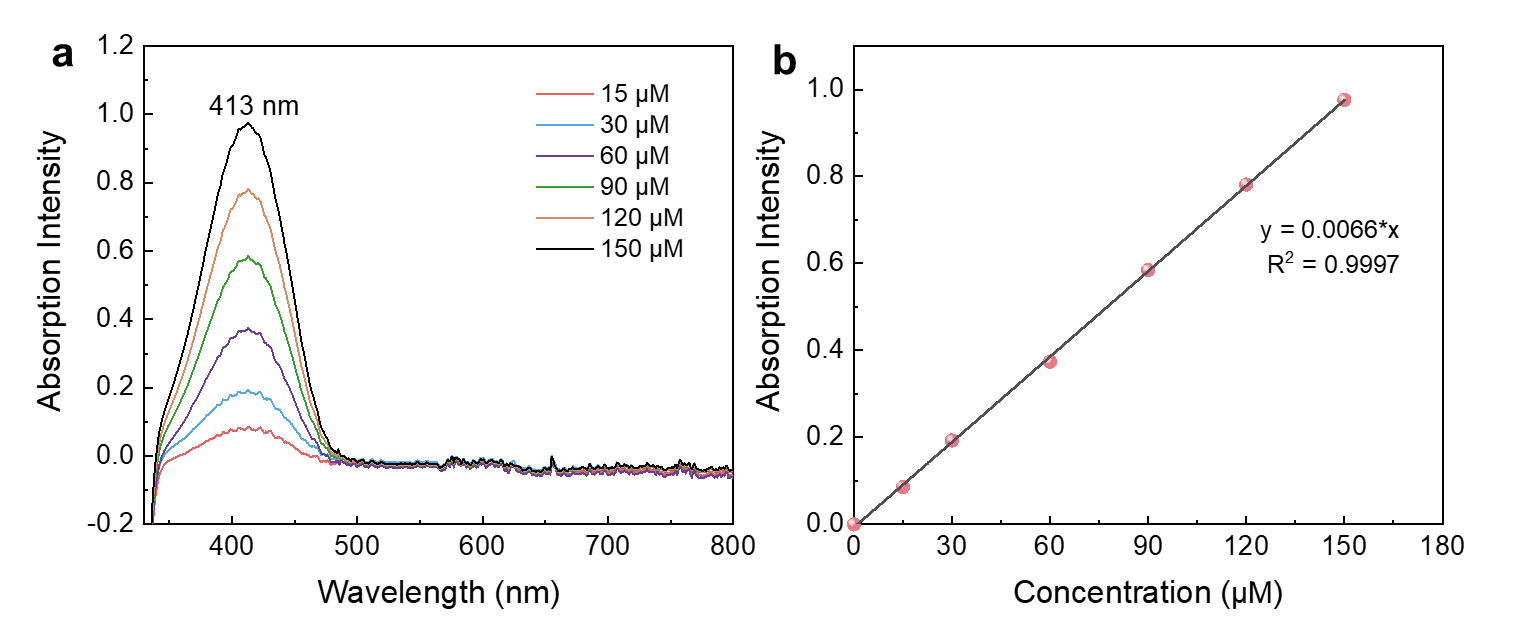


**Supplementary Fig. 12** **a** UV-vis absorption spectra of the product from the reaction between acetyl acetone and HCHO of different known concentrations. **b** Calibration curve obtained by plotting the HCHO concentration against absorption peak intensity at λ = 413 nm.


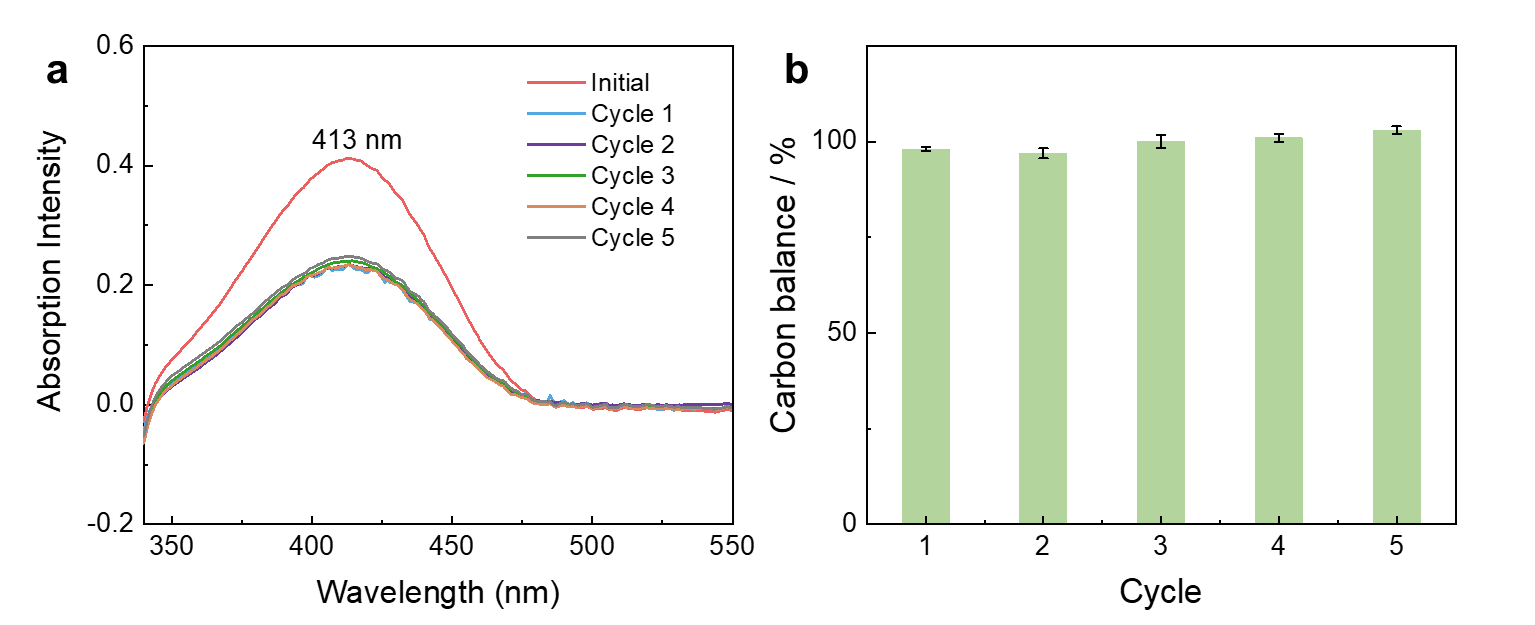


**Supplementary Fig. 13** **a** UV-vis absorption spectra of the testing solution containing acetyl acetone solution and the anolyte prior to and post per electrolysis cycle. **b** Carbon balance of HCHO oxidation in per electrolysis cycle. Error bars represent the standard deviation of at least three independent measurements. Condition: The electrolysis was investigated at five consecutive 1 h controlled-current electrolysis (150 mA) in a two-electrode electrolyzer using the Cu_3_Ag_7_/CF and Ni_3_N/Ni/NF couple but fresh electrolyte for each cycle, in which 1.0 M KOH was used as the catholyte and 1.0 M KOH with 0.6 M HCHO as the anolyte.


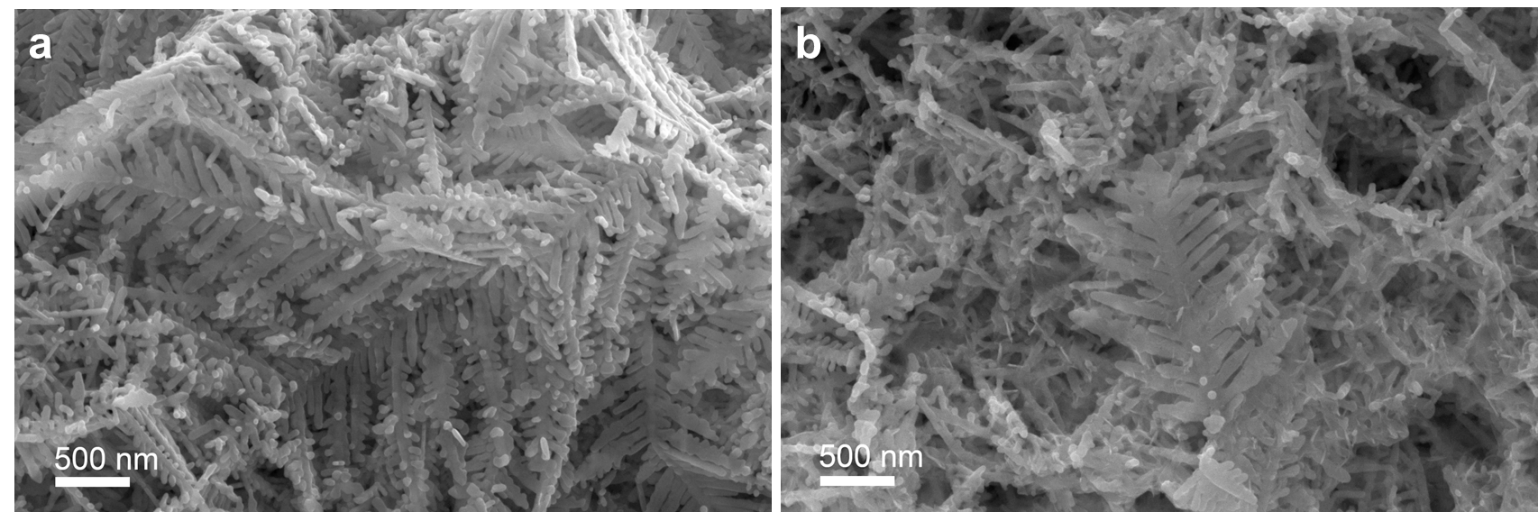


**Supplementary Fig. 14 a** SEM image of as-prepared Cu_3_Ag_7_/CF prior to electrolysis. **b** SEM of Cu_3_Ag_7_/CF after a 7.5 h chronoamperometry experiment at a cell voltage of 0.6 V in a two-electrode electrolyzer consisting of Cu_3_Ag_7_/CF anode and Ni_3_N/Ni/NF cathode, in which 1.0 M KOH was used as the catholyte and 1.0 M KOH with 0.6 M HCHO as the anolyte.

**
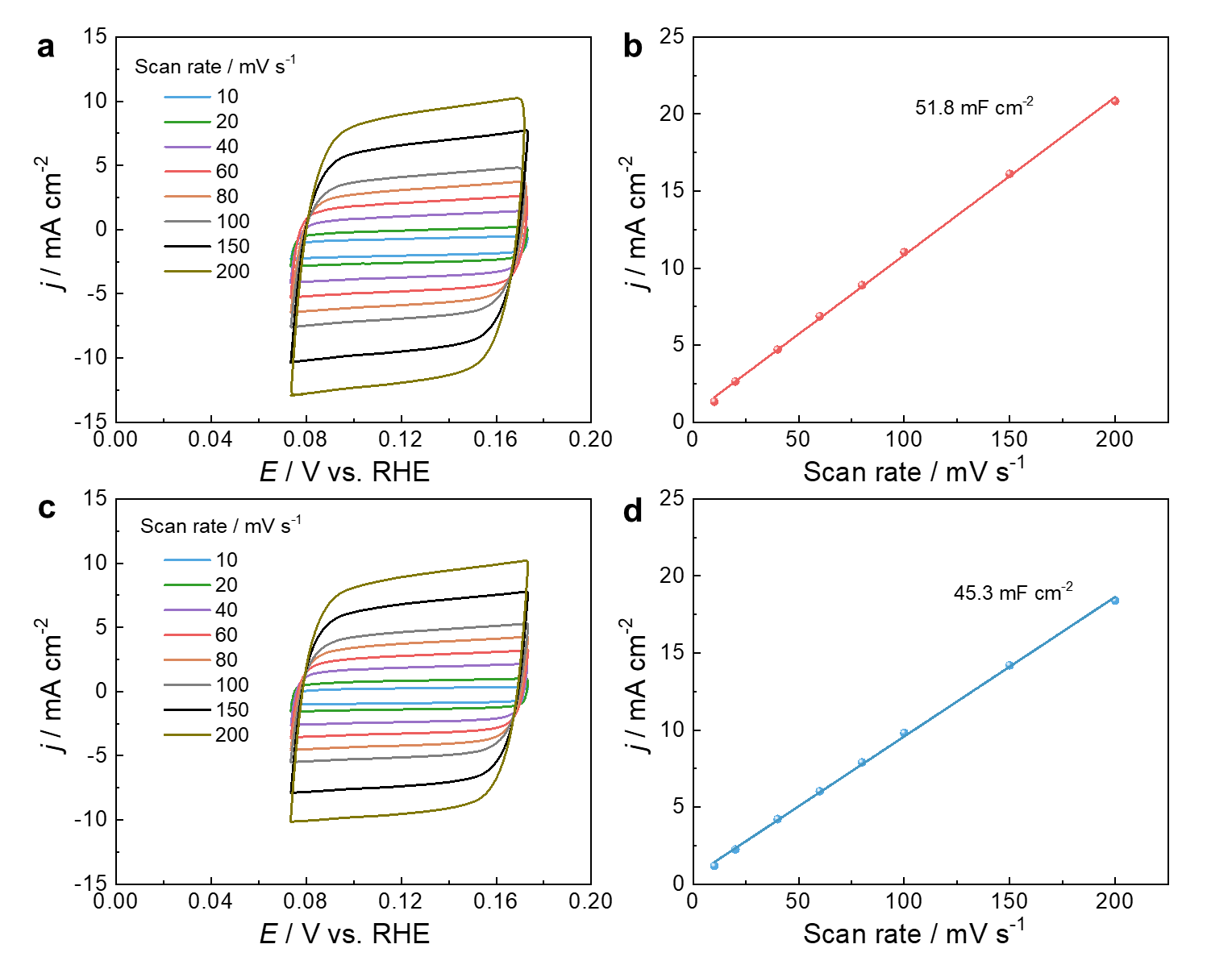
**

**Supplementary Fig. 15** CV curves for the Cu_3_Ag_7_/CF (**a**) before and (**c**) after chronoamperometric experiment were collected in a non-Faradaic region with various scan rates ranging from 20 to 200 mV s^-1^ at potentials between 0.07 V and 0.17 V vs。 RHE in 1.0 M KOH under Ar. Electrochemical double-layer capacitances (Cdl) values of Cu_3_Ag_7_/CF electrode (**b**) before and (**d**) after chronoamperometric experiment.


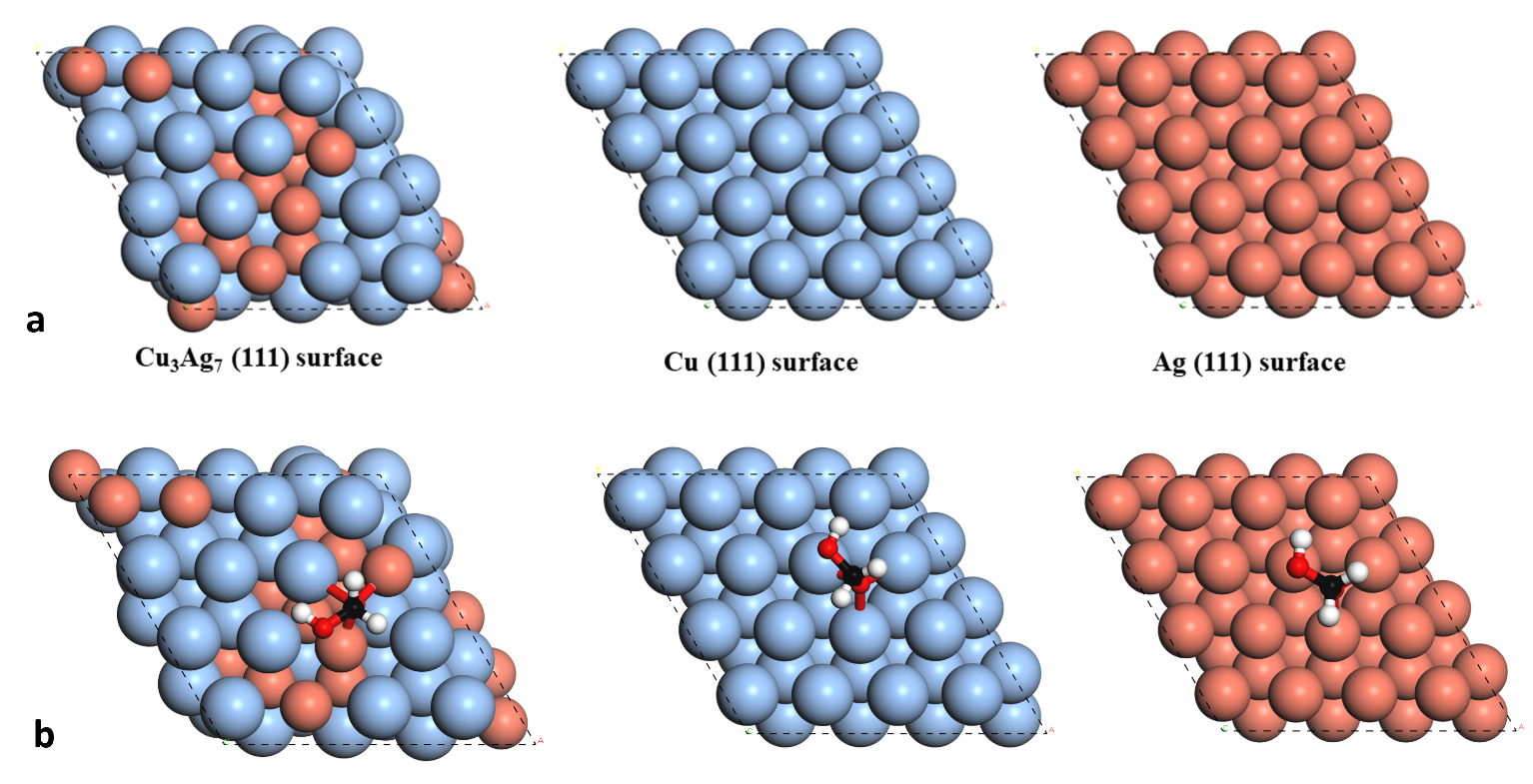


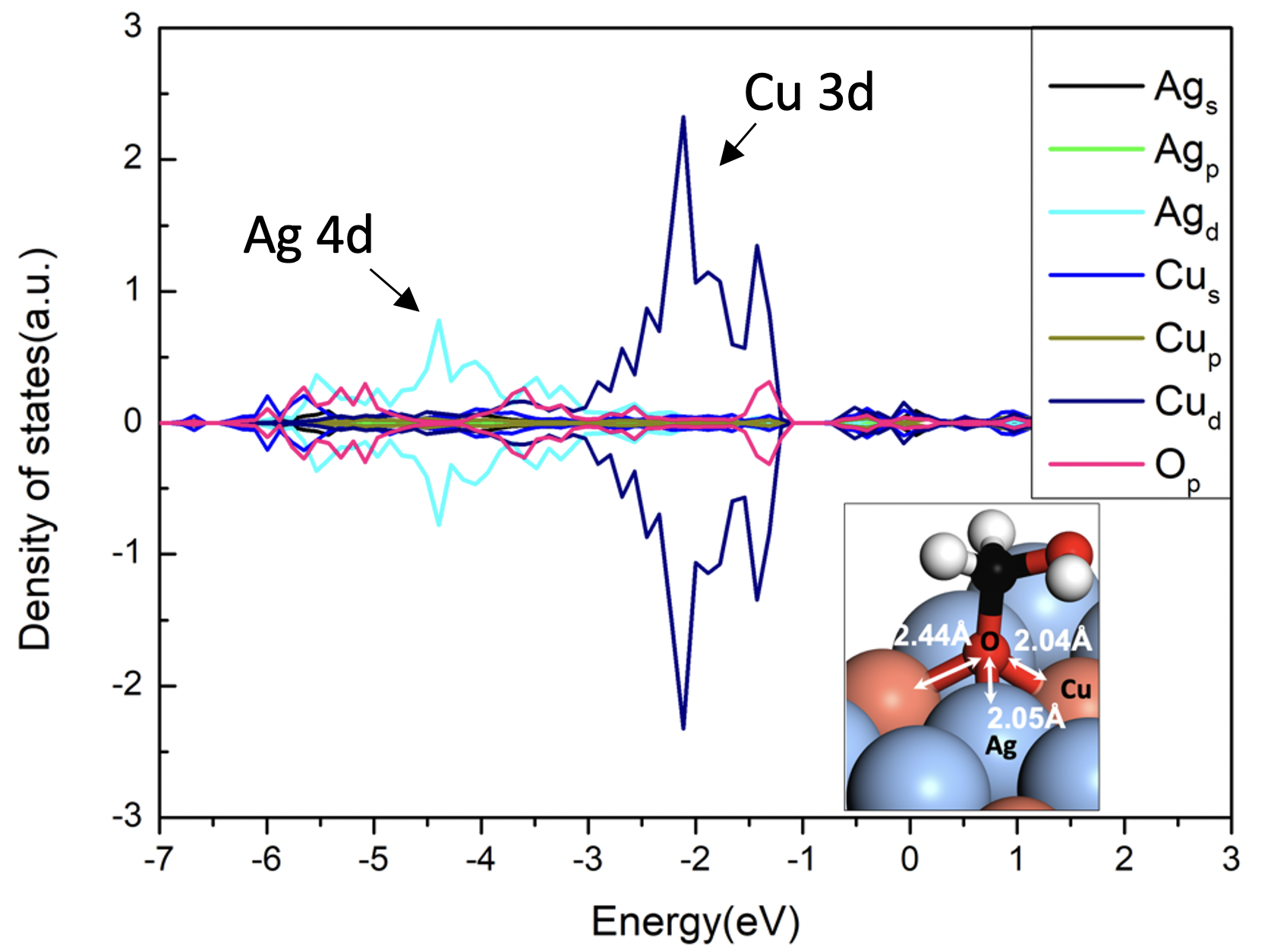


**c**

**Supplementary Fig. 16** **a** Models of the (111) surfaces of Cu_3_Ag_7_, Cu, and Ag. **b** Optimized geometry of adsorbed H_2_C(OH)O on the surfaces. **c** Projected density of states of the O atom of CH_2_OHO and the Cu and Ag atoms at the adsorption site (see inset for the atoms being analyzed; +/- y-values indicate spin up/down channels).


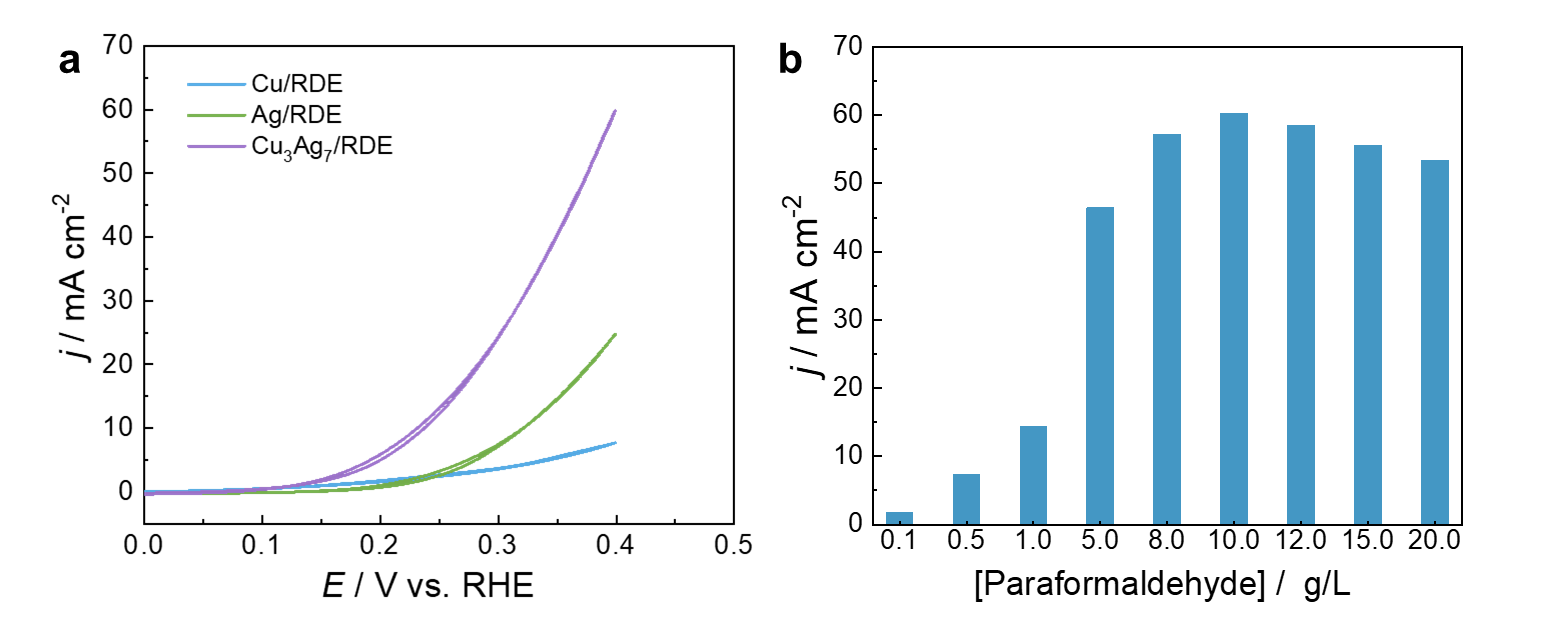


**Supplementary Fig. 17** **a** CV curves of Cu/RDE, Ag/RDE, and Cu_3_Ag_7_/RDE in 1.0 M KOH in the presence of 10.0 g/L PFA collected at 1500 rpm and 10 mV/s. **b** CV curves of Cu_3_Ag_7_/RDE in 1.0 M KOH in the presence of different mass concentrations of PFA (g/L) collected at 1500 rpm and 10 mV/s.


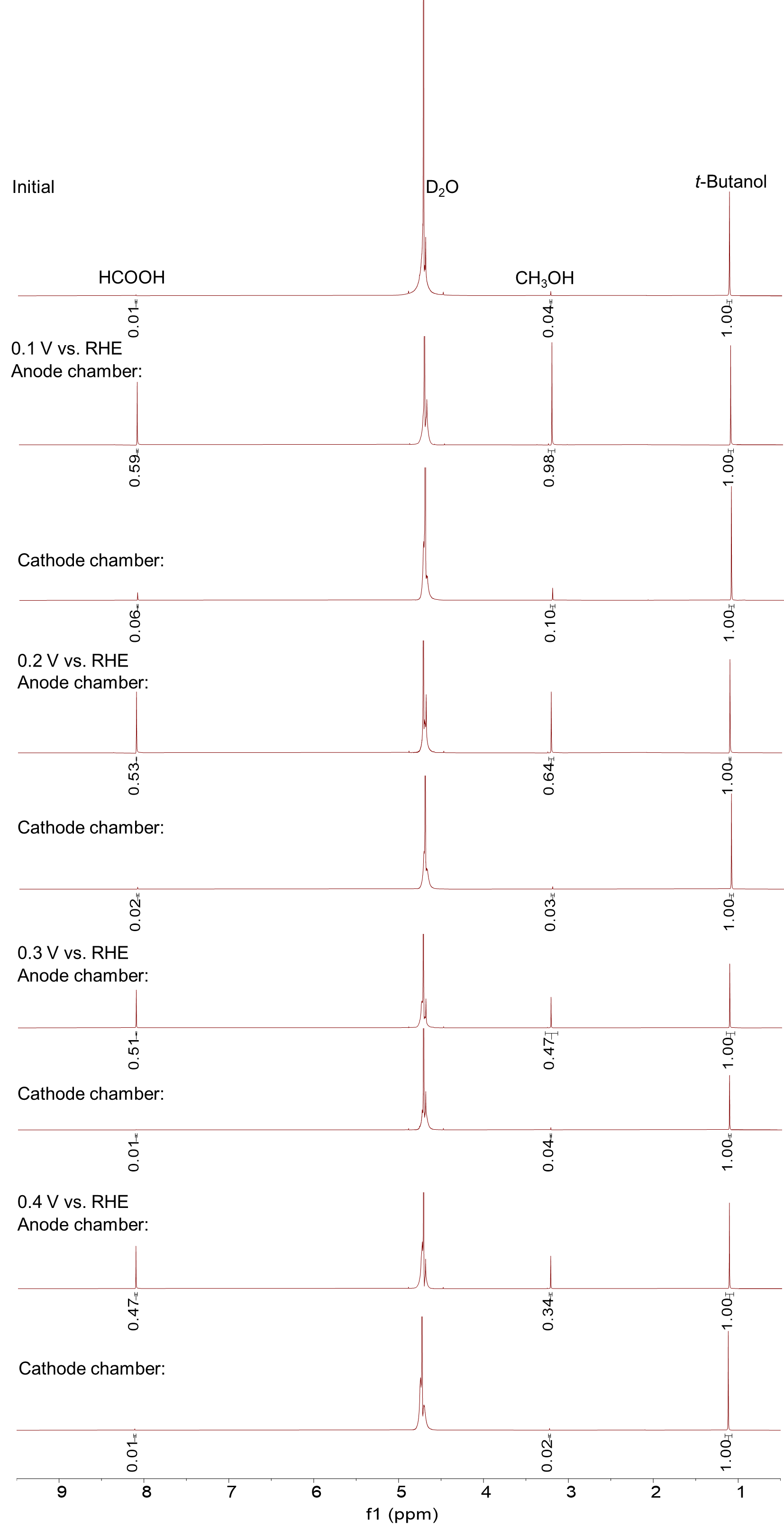


**Supplementary Fig. 18** The ^1^H NMR (D_2_O, 400 MHz) of organic products in the liquid phase of the anode and cathode chamber during each electrolysis with different voltage inputs (0.1 – 0.4 V_RHE_) in a two-compartment electrochemical cell using Cu_3_Ag_7_/CF as the working electrode, in which 1.0 M KOH was used as the catholyte and 1.0 M KOH with 10.0 g/L PFA as the anolyte.


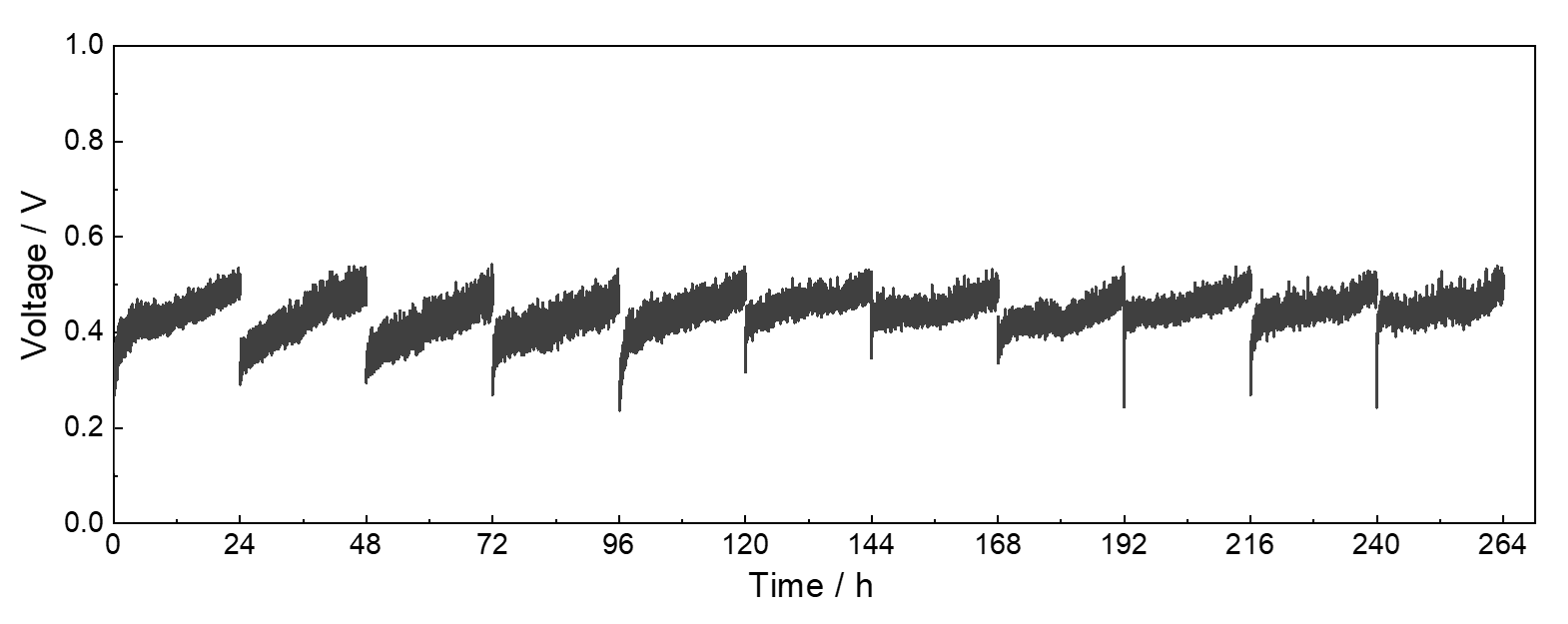


**Supplementary Fig. 19**. Chronopotentiometric curves for controlled-current electrolysis at 100 mA/cm^2^.

Condition: The electrolysis was carried out at controlled-current electrolysis (100 mA cm^-2^) in a two-electrode flow cell using the Cu_3_Ag_7_/CF and Ni_3_N/Ni/NF couple. The catholyte (1.0 M KOH) and anolyte (1.0 M KOH and 10.0 g/L PFA) in the tanks were fed into the cell at a flow rate of 50 mL min^−1^ and recycled in 24 h. The electrolyte was refreshed every 24 h.

**
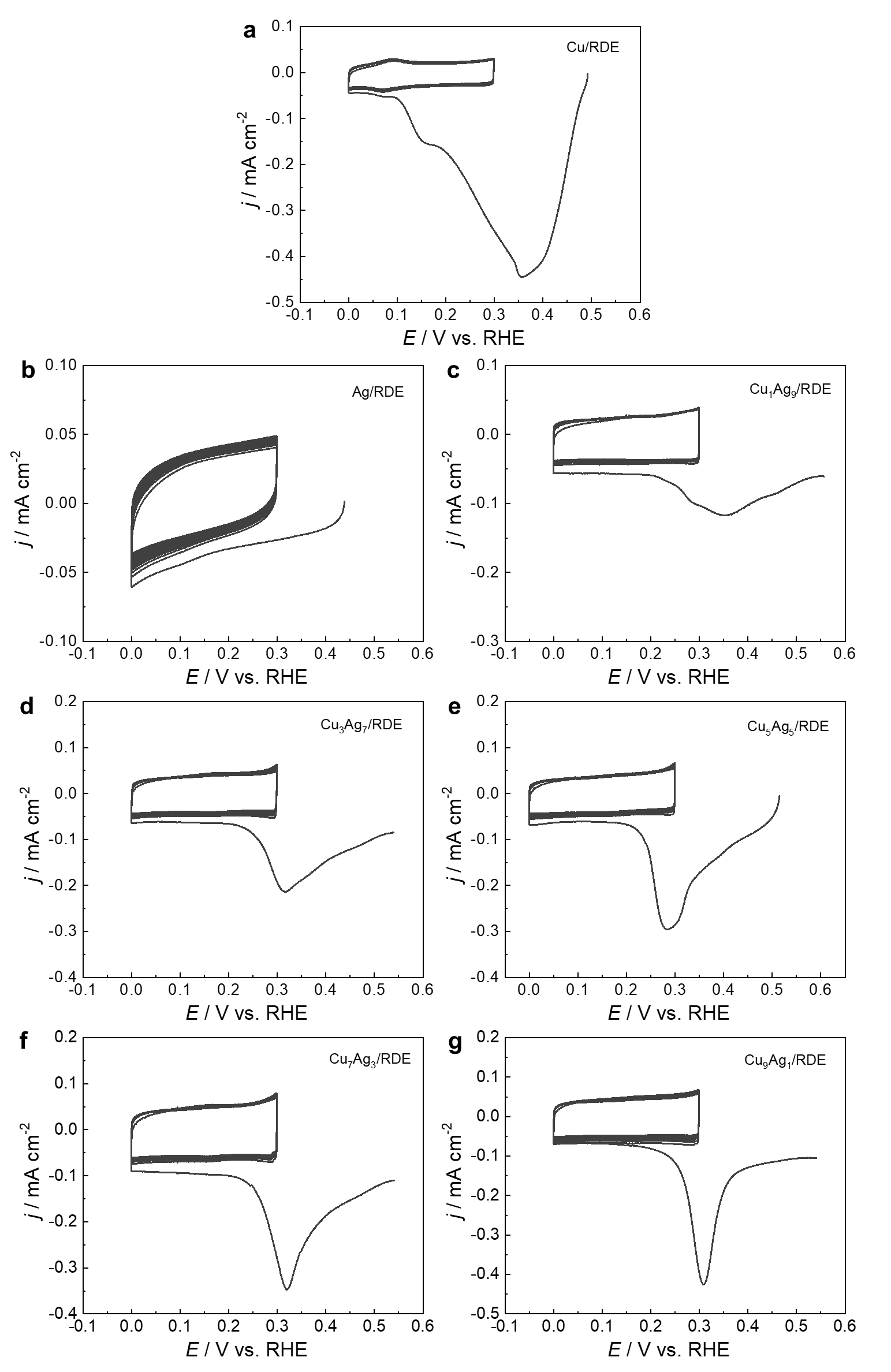
**

**Supplementary Fig. 20** (**a-g**) CV curves of Cu/RDE, Ag/RDE, and Cu_x_Ag_10-x_/RDE from 0 V_RHE_ to 0.3 V_RHE_ at 50 mV s^-1^ for 20 cycles in 1.0 M KOH under Ar.

**Supplementary Table 1** A comparison of electrolysis performance of inorganic and organic feedstocks electrooxidation integrated with HER in two-electrodes electrolyzer.

| **Oxidative**  **substrates** | **Catalysts** | **Anodic**  **product** | **Current**  **Density**  **(mA/cm^2^)** | **Cell voltage**  **(V)** | **References** |
| --- | --- | --- | --- | --- | --- |
| HCHO | Cu_3_Ag_7_/CF(+)\|\|Ni_3_N/Ni/NF(–) | H_2_, formate | 100  500 | 0.22  0.60 | **This work** |
| N_2_H_4_ | NiCo@C/MXene/CF(+, –) | N_2_ | 500 | 1.15 | 1 |
| N_2_H_4_ | Ni_2_P/NF(+, –) | N_2_ | 500 | 1.0 | 2 |
| Urea | NF/NiMoO^-^Ar(+)\|\|NF/NiMoO-H_2_(–) | N_2_, CO_2_ | 100 | 1.55 | 3 |
| Urea | MoP@NiCo-LDH/NF-20(+)\|\|MoP@NiCo-LDH/NF-20(–) | N_2_, CO_2_ | 100 | 1.405 | 4 |
| Na_2_S | CoS_2_@C/MXene/NF (+) \|\|  CoO@C/MXene/NF (–) | Sulfur | 300 | 0.97 | 5 |
| Ammonia | NiCu/CP(+)\|\|Pt/C(–) | N_2_ | 16 | 1.0 | 6 |
| Methanol | Co(OH)_2_@HOS/CP(+, –) | Formate | 70 | 1.66 | 7 |
| Methanol | Pt-Co_3_O_4_/CP(+, –) | Formate | 100 | 1.08 | 8 |
| Ethanol | Fmodified β-FeOOH(+)\|\|Pt/C(–) | Ethyl acetate | 10 | 1.43 | 9 |
| Glucose | Cu(OH)_2_/CF(+)\|\|Pt/C(–) | Glucaric acid | 100 | 0.92 | 10 |
| Glucose | NiFeO_x_-NF(+)\|\|NiFeN_x_-NF(–) | Glucaric acid | 200 | 1.48 | 11 |
| Benzyl alcohol | hp-Ni/NF(+, –) | Benzoic acid | 100 | 1.66 | 12 |
| Furfural | Ni_2_P/Ni/NF(+, –) | Furfural acid | 100 | 1.56 | 13 |
| HMF | Ni_2_P NPA/NF(+, –) | 2, 5-furandicarboxylic acid | 100 | 1.64 | 14 |
| HMF | Ni_2_S_3_/NF(+, –) | 2, 5-furandicarboxylic acid | 100 | 1.64 | 15 |

**Supplementary Table 2** The FE of anodic products and carbon balance based on the amounts of H_2_ and organic products during each electrolysis in a two-electrode electrolyzer.

| Entry | n_HCOOH_  (mmol) | | n_CH3OH_  (mmol) | n_HCOO-_  (mmol) | FE_HCOO-_ | n_H2_  (mmol) | FE_H2_ | n_HCHO_ （mmol） | Carbon balance |
| --- | --- | --- | --- | --- | --- | --- | --- | --- | --- |
|  | From  HCHO electrooxidation | From  Cannizzaro reaction | From  Cannizzaro reaction |  |  |  |  |  |  |
| Cycle 1 | 5.95 | 3.57 | 3.57 | (570C)  5.91 | 100% | 5.95 | 200% | 18.14 | 100% |
| Cycle 2 | 5.90 | 2.98 | 2.98 | (570C)  5.91 | 100% | 5.95 | 200% | 18.14 | 97% |
| Cycle 3 | 5.94 | 3.34 | 3.34 | (570C)  5.91 | 100% | 5.95 | 200% | 17.79 | 98% |
| Cycle 4 | 5.70 | 4.12 | 4.12 | (550C)  5.70 | 100% | 5.70 | 200% | 17.78 | 101% |
| Cycle 5 | 5.68 | 3.88 | 3.88 | (550C)  5.70 | 100% | 5.70 | 200% | 18.83 | 103% |

***Condition**: The electrolysis was carried out at five consecutive 1 h controlled-current electrolysis (150 mA) in a two-electrode electrolyzer using the Cu_3_Ag_7_/CF and Ni_3_N/Ni/NF couple but fresh electrolyte for each cycle, in which 1.0 M KOH was used as the catholyte and 1.0 M KOH with 0.6 M HCHO as the anolyte.

**Supplementary Table 3** The FE of anodic products based on the amounts of H_2_ and organic products during each electrolysis with different voltage inputs (0.1 – 0.4 V_RHE_) in a two-compartment electrochemical cell using Cu_3_Ag_7_/CF as the working electrode, in which 1.0 M KOH was used as the catholyte and 1.0 M KOH with 10.0 g/L paraformaldehyde as the anolyte.

| Voltage input | n_HCOOH_  (mmol) | | n_CH3OH_  (mmol) | n_HCOO-_  (mmol) | FE_HCOO-_ | n_H2_  (mmol) | FE_H2_ |
| --- | --- | --- | --- | --- | --- | --- | --- |
|  | From HCHO electrooxidation | From  Cannizzaro reaction | From  Cannizzaro reaction |  |  |  |  |
| 0.4 V_RHE_ for 1.0 h | 4.12 | 0.71 | 0.76 | (400C)  4.14 | 100% | 2.08 | 100% |
| 0.3 V_RHE_ for 1.2 h | 4.18 | 1.05 | 1.05 | (400C)  4.14 | 100% | 2.08 | 100% |
| 0.2 V_RHE_ for 2.0 h | 4.15 | 1.40 | 1.40 | (400C)  4.14 | 100% | 2.08 | 100% |
| 0.1 V_RHE_ for 3.8 h | 4.19 | 2.37 | 2.37 | (400C)  4.14 | 100% | 2.08 | 100% |

**References**

1. Sun, F.*, et al.* Energy-saving hydrogen production by chlorine-free hybrid seawater splitting coupling hydrazine degradation. *Nat. Commun.* **12**, 4182 (2021).

2. Tang, C.*, et al.* Energy-saving electrolytic hydrogen generation: Ni_2_P nanoarray as a high-performance non-noble-metal electrocatalyst. *Angew. Chem. Int. Ed.* **56**, 842-846 (2017).

3. Yu, Z.-Y.*, et al.* Ni–Mo–O nanorod-derived composite catalysts for efficient alkaline water-to-hydrogen conversion via urea electrolysis. *Energy Environ. Sci.* **11**, 1890-1897 (2018).

4. Wang, T., Wu, H., Feng, C., Zhang, L. & Zhang, J. MoP@NiCo-LDH on nickel foam as bifunctional electrocatalyst for high efficiency water and urea–water electrolysis. *J. Mater. Chem. A* **8**, 18106-18116 (2020).

5. Zhang, L., Wang, Z. & Qiu, J. Energy-saving hydrogen production by seawater electrolysis coupling sulfion degradation. *Adv. Mater.* **34**, 2109321 (2022).

6. Xu, W.*, et al.* Electrodeposited NiCu bimetal on carbon paper as stable non-noble anode for efficient electrooxidation of ammonia. *Appl. Catal. B: Environ.* **237**, 1101-1109 (2018).

7. Xiang, K.*, et al.* Boosting H_2_ generation coupled with selective oxidation of methanol into value‐added chemical over cobalt hydroxide@hydroxysulfide nanosheets electrocatalysts. *Adv. Funct. Mater.* **30**, 1909610 (2020).

8. Xiang, K.*, et al.* Bifunctional Pt–Co_3_O_4_ electrocatalysts for simultaneous generation of hydrogen and formate via energy-saving alkaline seawater/methanol co-electrolysis. *J. Mater. Chem. A* **9**, 6316-6324 (2021).

9. Chen, G.-F., Luo, Y., Ding, L.-X. & Wang, H. Low-voltage electrolytic hydrogen production derived from efficient water and ethanol oxidation on fluorine-modified FeOOH anode. *ACS Catal.* **8**, 526-530 (2017).

10. Zhang, Y.*, et al.* Coupling glucose-assisted Cu(I)/Cu(II) redox with electrochemical hydrogen production. *Adv. Mater.* **33**, 2104791 (2021).

11. Liu, W. J.*, et al.* Efficient electrochemical production of glucaric acid and H_2_ via glucose electrolysis. *Nat. Commun.* **11**, 265 (2020).

12. You, B., Liu, X., Liu, X. & Sun, Y. Efficient H_2_ evolution coupled with oxidative refining of alcohols via a hierarchically porous nickel bifunctional electrocatalyst. *ACS Catal.* **7**, 4564-4570 (2017).

13. Jiang, N., Liu, X., Dong, J., You, B., Liu, X. & Sun, Y. Electrocatalysis of furfural oxidation coupled with h2 evolution via nickel-based electrocatalysts in water. *ChemNanoMat* **3**, 491-495 (2017).

14. You, B., Jiang, N., Liu, X. & Sun, Y. Simultaneous H_2_ generation and biomass upgrading in water by an efficient noble-metal-free bifunctional electrocatalyst. *Angew. Chem. Int. Ed.* **55**, 9913-9917 (2016).

15. You, B., Liu, X., Jiang, N. & Sun, Y. A general strategy for decoupled hydrogen production from water splitting by integrating oxidative biomass valorization. *J. Am. Chem. Soc.* **138**, 13639-13646 (2016).
