# Supplementary material for: Dual hydrogen production from electrocatalytic water reduction coupled with formaldehyde oxidation via a copper-silver electrocatalyst
Source: Nat Commun. 2023 Jan 31;14:525. doi: 10.1038/s41467-023-36142-7 (PMC9889775; doi:10.1038/s41467-023-36142-7)
Supplement: Supplementary file 3 — Description of Additional Supplementary Files [file 41467_2023_36142_MOESM3_ESM.docx]

Description of additional supplementary information

Supplementary Data 1: Optimized bulk and (111) surface structures in VASP POSCAR format.
